# Iron Complexes as Potential Carriers of Diffuse Interstellar Bands: The Photodissociation Spectrum of Fe^+^(H_2_O) at Optical Wavelengths

**DOI:** 10.1021/acs.jpca.4c00148

**Published:** 2024-02-13

**Authors:** Marcos Juanes, Shan Jin, Rizalina T. Saragi, Christian van der Linde, Alexander Ebenbichler, Norbert Przybilla, Milan Ončák, Martin K. Beyer

**Affiliations:** † Institut für Ionenphysik und Angewandte Physik, Universität Innsbruck, Technikerstraße 25, Innsbruck 6020, Austria; ‡ Dept. Química Física y Química Inorgánica, University of Valladolid, Paseo de Belén 7, Valladolid 47011, Spain; § Institut für Astro- und Teilchenphysik, Universität Innsbruck, Technikerstr. 25/8, Innsbruck 6020, Austria

## Abstract

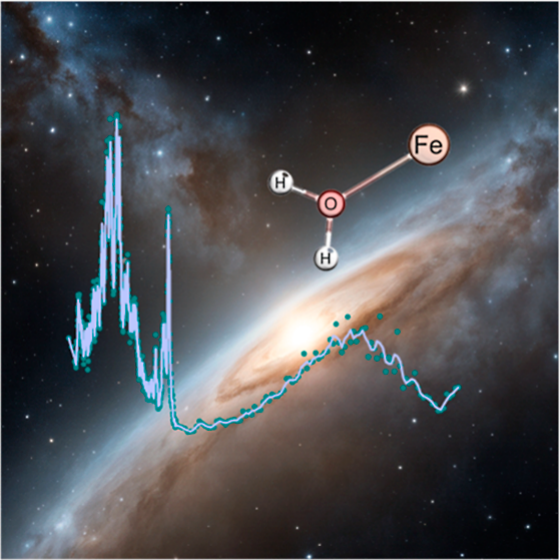

The fullerene ion C_60_^+^ is the only carrier of diffuse interstellar bands (DIBs) identified so far. Transition-metal compounds feature electronic transitions in the visible and near-infrared regions, making them potential DIB carriers. Since iron is the most abundant transition metal in the cosmos, we here test this idea with Fe^+^(H_2_O). Laboratory spectra were obtained by photodissociation spectroscopy at 80 K. Spectra were modeled with the reflection principle. A high-resolution spectrum of the DIB standard star HD 183143 served as an observational reference. Two broad bands were observed from 4120 to 6800 Å. The 4120–4800 Å band has sharp features emerging from the background, which have the width of DIBs but do not match the band positions of the reference spectrum. Calculations show that the spectrum arises from a d–d transition at the iron center. While no match was found for Fe^+^(H_2_O) with known DIBs, the observation of structured bands with line widths typical for DIBs shows that small molecules or molecular ions containing iron are promising candidates for DIB carriers.

## Introduction

Diffuse interstellar bands (DIBs) are absorption features that are imprinted on the spectra of background sources by the diffuse interstellar medium (ISM), with the first detection dating back to 1922.^1^ The DIBs remained an important mystery to a large extent over the past century.^2^ They are ubiquitous not only toward reddened sight lines in the Milky Way but also toward sources in dust-harboring nearby galaxies^3−5^ and even out to cosmological distances.^6^ About 600 DIBs have been detected over the past century,^7^ with the most recent detections being made in the near-infrared.^8,9^ The focus on laboratory spectroscopy to find DIB carriers has been put on carbon compounds, mostly polycyclic aromatic hydrocarbons (PAHs), fueled by the identification of C_60_^+^ as the only known DIB carrier to date.^10,11^ The identification of DIBs in the near-infrared region, however, requires ever larger molecules to afford low-lying electronic transitions. As argued recently by Kappe et al.,^12^ larger PAHs are difficult to form and tend to become more reactive with size, which reduces their overall abundance.

So far, only four DIBs have an unambiguously identified carrier, the Buckminster-Fullerene cation C_60_^+^.^10,11,13^ In general, the carriers are supposed to be large carbon-based molecules (∼10–100 atoms) in the gas phase, such as (long) carbon chains, PAHs, or fullerenes.^14,15^ Photochemical processing of large PAHs appears to directly form C_60_ via a top-down route,^16^ along with other carbon compounds like cages, tubes, or bowls.^17^ Only carbon chemistry appears to provide the complexity to produce the plethora of observed absorption features of different widths, depths, and line profiles, with carbonaceous dust and C^+^ contributing additional ingredients for the production and growth of DIB carriers. Given the large number of potential candidates, further identifications of carriers have been unsuccessful to date.^18^ However, the open 3d shells of the transition metals titanium to nickel provide a different possibility to realize complex spectra, even for relatively simple molecules. These elements are largely depleted from the gas phase onto dust grains in the cold ISM,^19,20^ and potentially onto DIB carriers.

In the laboratory, transition-metal complexes are mostly studied in solution, where the interaction of the solvent leads to very broad, structureless absorptions. In the gas phase, however, photodissociation spectra have been obtained with vibrational resolution, for example, for V^+^(H_2_O) as discussed by Lessen et al.,^21^ which exhibits a progression of vibrational bands starting at 15,880 cm^–1^, showing partially resolved rotational structure with isolated peaks that are 2 cm^–1^ wide. The availability of vanadium in the ISM is comparatively limited, but iron as the most abundant transition metal in the universe is a significant component of interstellar dust particles.^22−24^ Among iron-bearing compounds, FeO^25,26^ and FeCN^27^ have been identified in the ISM. In the laboratory, rotational lines of Fe^+^(CO) have been measured by Halfen and Ziurys^28^ to aid identification of this compound in molecular clouds. These authors pointed out that molecular ions could be hidden carriers of metallic elements in such clouds. A predissociative state of FeO^+^ was measured by Metz and co-workers,^29^ as well as two vibrational progressions of a state below the dissociation limit.^30^ Pioneering work by Cassady and Freiser established the photodissociation thresholds of FeOH^+^, Fe^+^(CO), and Fe^+^(C_2_H_4_).^31^ Duncan and co-workers found very broad photodissociation spectra for Fe^+^(C_2_H_2_) and Fe^+^(C_6_H_6_)_1,2_ complexes,^32,33^ which nevertheless exhibit some structure in the photodissociation continuum. On the basis of the continuous spectrum, however, these authors concluded that iron-benzene complexes cannot be DIB carriers,^33^ which was previously suggested by Lanza, Simon, and Ben Amor.^34^

The presence of the FeH^+^ ion has been postulated since the 1980s,^35,36^ but due to the lack of laboratory data, it has not been identified so far in the ISM. Quantum chemical calculations are rather difficult due to the complex electronic structure involving a multitude of spin–orbit coupling states.^37−39^ We have recently reported the infrared spectrum of Ar_2_FeH^+^ as a first experimental characterization of the Fe–H^+^ stretching mode.^40^ Overall, more laboratory measurements of gas-phase molecules or molecular ions containing transition metals as potential carriers of DIBs are required to test this hypothesis.

Water is abundant in some regions of the ISM in form of ice particles and also in the gas phase.^41^ At the low temperatures in the ISM, formation of Fe^+^(H_2_O) by radiative association can be efficient since the complex has already six internal degrees of freedom, and H_2_O is an excellent infrared emitter. The binding energy of H_2_O to Fe^+^ is 1.33 ± 0.05 eV, as measured by Schultz and Armentrout.^42^ These arguments render the presence of Fe^+^(H_2_O) in the ISM plausible; thus, it seems to be a good candidate as a DIB carrier. The infrared spectra of the argon-tagged complex Fe^+^(H_2_O)Ar_1,2_ were measured in the O–H stretch region by Walters and Duncan.^43^ Density functional calculations on the B3LYP/DZVP level of theory predicted a quartet ground state with *C*_2v_ symmetry.^44^ We, therefore, investigated the spectral properties of this complex by photodissociation spectroscopy in the 4100–6800 Å region.

## Experimental and Computational Methods

The experiments were performed on a modified 4.7 T Fourier-Transform Ion Cyclotron Resonance (FT-ICR) Bruker/Spectrospin CMS47X mass spectrometer equipped with an external laser vaporization ion source.^45^ Iron–water complexes were obtained by laser vaporization^46−48^ (Nd/YLF, 527 nm) of a solid iron target made from enriched ^56^Fe (STB Isotope Germany GmbH), followed by a supersonic jet expansion in a He + H_2_O carrier gas mixture and guided to the center of the ICR cell.^49^ There they were stored and irradiated for 10 s, followed by recording a mass spectrum.^50^ In the supersonic expansion, the ionic complexes were cooled to internal energies below room temperature. To minimize heating by ambient blackbody radiation, the cell was cooled to 80 K with liquid nitrogen.^51^ Tuneable monochromatic light was generated by an EKSPLA NT342B Optical Parametric Oscillator (OPO) laser system operating at a 20 Hz pulse repetition rate and covering the 4120–6800 Å region.^52^ The wavelength was calibrated with a SHR high–resolution wide-range spectrometer (SOLAR Laser Systems), determining the line width as <0.5 Å in agreement with specifications.

A high-quality spectrum of the DIB standard star HD 183143 was observed with the fiber-fed extended range optical spectrograph,^53^ on the ESO/Max Planck 2.2 m telescope in La Silla in Chile on August 18, 2013. The fully reduced data were downloaded from the ESO Data Portal^a^ normalized and corrected for cosmics and for stellar lines in the wavelength regions of interest here.

Quantum chemical calculations in the electronic ground state were performed using coupled cluster singles and doubles methods (CCSD), along with the aug-cc-pVTZ basis sets; the wave function stability was tested prior to optimization. To obtain more reliable reaction energies, a single-point calculation with noniteratively included triples, CCSD(T)/aug-cc-pVQZ, was performed in optimized structures. For electronically excited states, complete active space–self-consistent field (CASSCF) and multireference configuration interaction with the Davidson correction (MRCI + Q) were employed, along with the aug-cc-pVDZ basis set. Spin–orbit coupling was included through the Breit-Pauli Hamiltonian, employing CASSCF spin–orbit matrix elements and MRCI + Q energies. Coupled cluster calculations were performed in Gaussian,^54^ multireference calculations in Molpro.^55,56^

We analyzed the sensitivity of electronic energies of sextet and quartet states to the active space size using MRCI calculations in active spaces from (7,6) to (9,11) and (7,11) and CASSCF calculations with active spaces from (7,11) to (7,16) and (9,15). While the energies of the lowest sextet states are reasonably converged already with the minimal active space of (7,6), the quartet states are deeply influenced by the size of the active space, notably, the 4^4^B_1_ and 4^4^B_2_ states in the experimental window. As a compromise, the (7,11) active space was employed. For sextet and quartet states, 5 sextet and 30 quartet terms were included in the CASSCF calculation. The position of doublet states and higher-lying sextet states was evaluated by including 10 sextet and 12 doublet terms.

Due to the complicated electronic structure of the Fe^+^(H_2_O) ion, several approximations were introduced for efficient modeling of the molecular spectrum, employing the reflection principle.^57^ Within this approximation, the vibrational resolution of the spectra is lost. Sampling of the potential energy surface is performed within a harmonic approximation employing Monte Carlo integration of points arising from the Wigner distribution. The respective minimum structure and vibrational frequencies of the Fe^+^(H_2_O) ion were calculated at the CCSD/aug-cc-pVTZ level of theory, corresponding to ^4^B_2_ and ^6^A_2_ molecular terms. Note that further terms of other irreducible representations lie close in energy. Only vibrational degrees of freedom of A_1_ irreducible representation were considered, that is three out of six vibrational degrees of freedom, corresponding to symmetric O–H stretch, H_2_O bending, and Fe–OH_2_ stretch. The spectra were modeled using 200 points sampled from the Wigner distribution. In each point, an MRCI + Q(7,11) calculation including spin–orbit coupling was performed with 5 sextet terms and 20 quartet ones; Gaussian broadening of calculated transitions with a full width at half-maximum of 0.05 eV was employed. A certain contribution of excitations from higher-lying electronic states can be expected, estimated below 10%; however, the respective spectra are qualitatively similar, with only a slight red-shift, not influencing the overall spectral shape. The spectrum of the sextet-quartet transitions is shifted by −0.15 eV to account for the CCSD(T)/aug-cc-pVQZ//CCSD/aug-cc-pVDZ prediction that sextet and quartet ground states lie at the same energy (sextet is calculated to be preferred by 0.23 eV when the Douglas-Kroll-Hess fourth-order approach is used to include relativistic effects). Due to the multitude of technical parameters and the mentioned approximations, the results should be considered semiquantitative.

## Results and Discussion

### Experimental Photodissociation Spectrum

**Figure 1:**
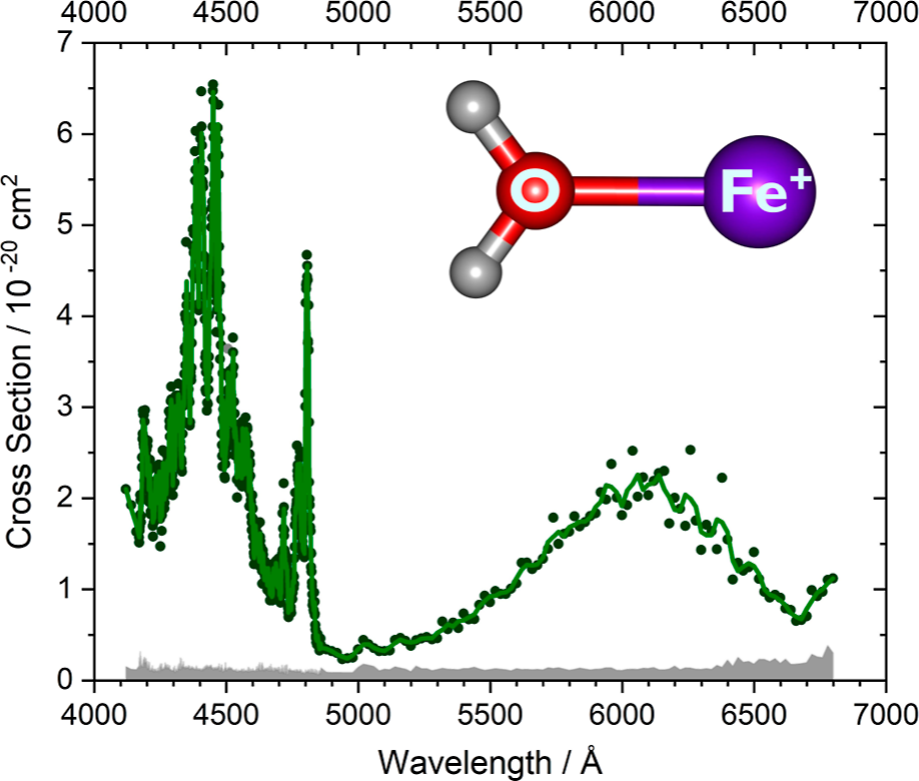
Photodissociation spectrum of Fe^+^(H_2_O) covering the 4120–6800 Å range, at 1 Å resolution below 4860 and 20 Å resolution above (dark green dots) and an adjacent average of the data points with a three-points window (green trace). The detection limit in the range of 10^–21^ cm^2^ is indicated (gray shaded area). The inset shows the molecular structure of the Fe^+^(H_2_O) cation.

The complete measured photodissociation spectrum, as shown in Figure 1, is obtained by irradiating the complex of Fe^+^(H_2_O), which was previously generated by standard laser vaporization and stored in the ICR cell at a temperature of 80 K. Photodissociation resulted in loss of H_2_O. Mass spectra were recorded in steps of 1 Å between 4120 and 4860 Å, the region where a richly structured spectrum is observed, and 20 Å between 4860 and 6800 Å, which corresponds to a broad absorption with little structure. The photon energy at 6800 Å is 1.82 eV, well above the dissociation energy of the complex. Two broad absorptions were observed, from 4100–5000 to 5000–6700 Å. The higher-energy band exhibits a pronounced structure on top of the broad continuum. The section at 4480–4700 Å is relatively smooth, while three intense bands are present from 4700 to 4850 Å. In the low-energy band, with absorption cross sections around 10^–20^ cm^2^, some structure appears from 5900 to 6400 Å but not as pronounced as in the higher energy band. In view of the binding energy of 1.33 ± 0.05 eV,^42^ here calculated as 1.41 eV, the excited states responsible for the spectrum of Fe^+^(H_2_O) lie well above the dissociation asymptote to the fragments in their electronic ground states. The sharp features, however, imply that the initial absorption occurs into bound states, which after some time undergo internal conversion or intersystem crossing either to repulsive states, which would lead to immediate dissociation, or to lower-lying bound states, which afford dissociation by the redistribution of vibrational energy into the Fe^+^–OH_2_ dissociative coordinate.

Previously studied metal–water complexes, e.g. Co^+^(H_2_O),^58^ exhibited vibrationally resolved photodissociation spectra. This raises the question of why our Fe^+^(H_2_O) spectrum exhibits such a broad continuum. We were previously able to obtain vibrational resolution of one band in Mg^+^(H_2_O),^52^ under similar conditions as the current experiment. The temperature of 80 K certainly causes some thermal broadening, but the loss of the vibrational structure may also be caused by the photodissociation dynamics. If a sufficiently high number of electronically excited state is involved, photodissociation spectra may get completely continuous already for diatomic molecules cooled in a supersonic beam, as shown by Morse and co-workers.^59^ For the Fe^+^(H_2_O) with six internal degrees of freedom, the conditions for a continuous spectrum could be reached with a much smaller number of excited states as for a diatomic. In such a scenario, the photodissociation spectrum would exhibit a broad continuum even at the temperature of the interstellar microwave background. If the Fe^+^(H_2_O) complex with such a spectrum was present in the ISM, the broad absorptions would probably merge into the observational baseline, while the sharp features would emerge as potential DIBs.

### Observational DIB Spectra

**Figure 2:**
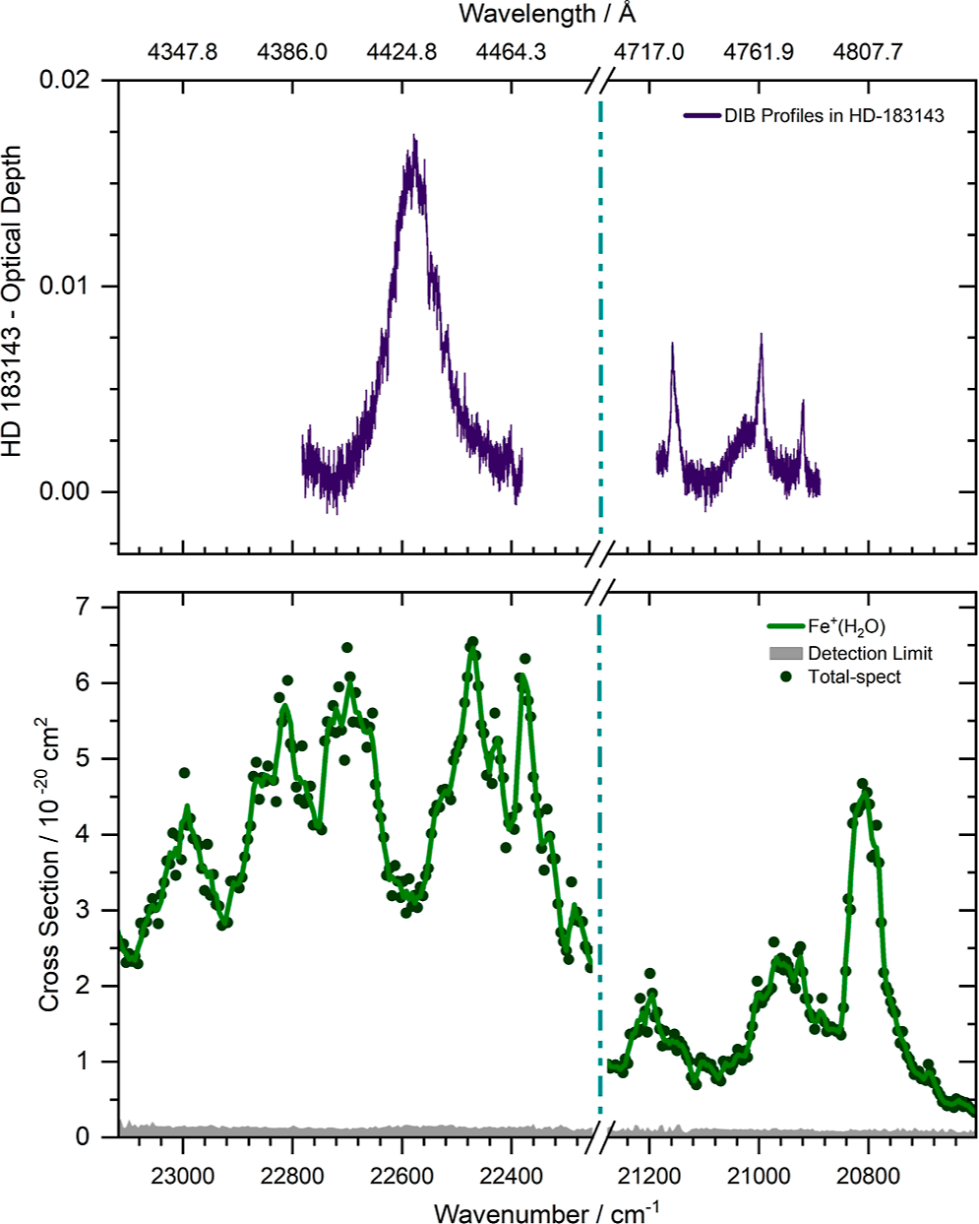
Measured photodissociation spectrum of Fe^+^(H_2_O) (*lower panel*, green, see Figure 1 for further details) versus observed DIB profiles in HD 183143 (*upper panel*, violet, displayed is the optical depth, making the observed absorption features more easily comparable to the laboratory measurements). The observed DIB profiles were corrected for stellar absorption lines.

For a comparison of our measurements with known DIBs, a high-quality spectrum with resolving power *R* = λ/Δλ = 48,000 and signal-to-noise ratio of *S*/*N* ≈ 190 at 4460 Å of the DIB standard star HD 183143 was considered. Figure 2 shows the most prominent features of the Fe^+^(H_2_O) spectrum together with the DIBs along the sightline toward HD 183143 (the observed absorption features are expressed here in terms of optical depth). A synthetic spectrum with individually optimized abundances based on the model parameters of Weßmayer et al.^60^ was employed for removing stellar absorption features, see Ebenbichler et al.^8^ for details of the methodology. The laboratory data lie in the wavelength region where DIBs are observed and also exhibit rotational envelopes that resemble some DIBs, but it is clearly far from a perfect match. Fe^+^(H_2_O) thus can be ruled out as a carrier for these bands. Apart from the obvious mismatch of the band positions, the relatively small absorption cross section in the range of 10^–20^ cm^2^ is also an argument against Fe^+^(H_2_O) as a DIB carrier. Strong absorptions with cross sections in the 10^–17^ to 10^–16^ cm^2^ range are obviously easier to detect. Carriers with much smaller cross sections must be present in a significantly higher abundance.

### Quantum Chemical Modeling

**Figure 3:**
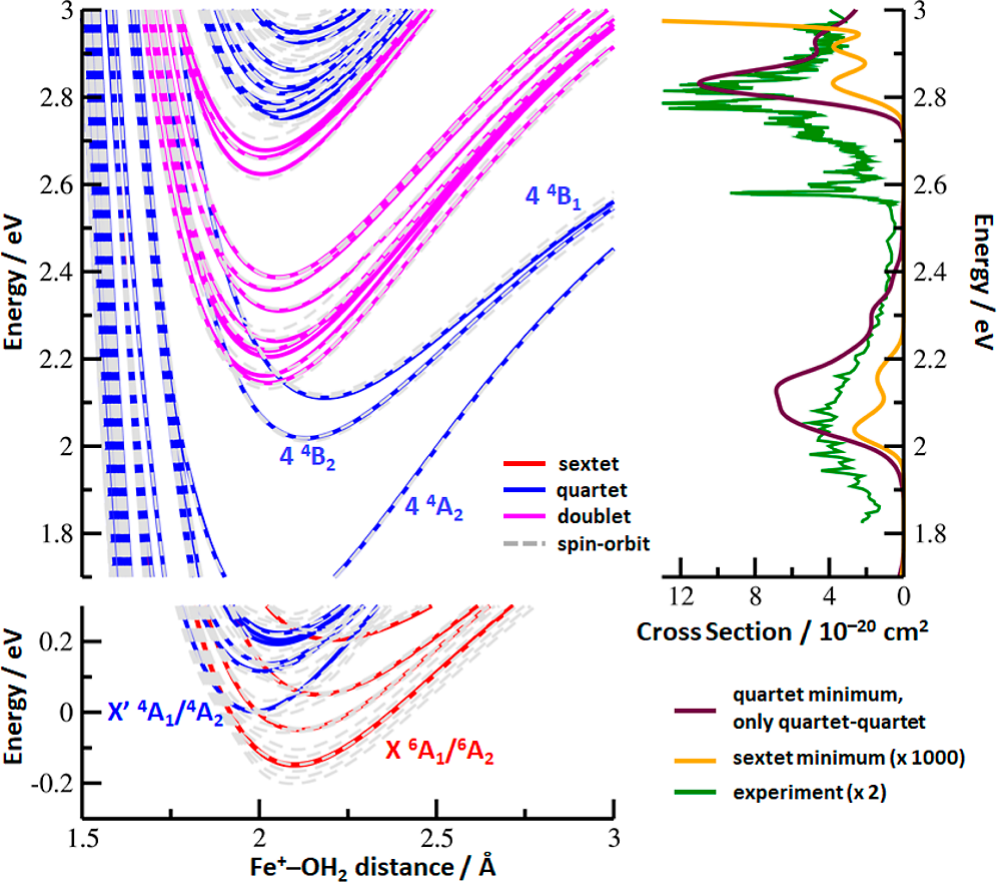
*Left panel*: potential energy curves for selected electronic states of Fe^+^(H_2_O) along the Fe^+^···H_2_O coordinate, with other structure parameters used as optimized at the CCSD/aug-cc-pVTZ level for quartet spin multiplicity. Splines are used to guide the eye. *Right panel*: simulated absorption spectra using the reflection principle for quartet–quartet transitions in the quartet minimum and sextet-quartet transitions in the sextet minimum. Calculated at the MRCI + Q(7,11)/aug-cc-pVDZ level of theory. The spectrum for the sextet-quartet transitions was shifted by −0.15 eV to match the relative stability of quartet/sextet as calculated at the CCSD(T)/aug-cc-pVQZ level (see the text).

Quantum chemical calculations of the Fe^+^(H_2_O) complex at the MRCI + Q/aug-cc-pVDZ level of theory predict a multitude of electronic states within the experimental energy range, in particular, after including spin–orbit coupling. Figure 3 presents a simplified picture of the most relevant potential curves along the *r*(Fe–O) dissociation coordinate. The calculated electronic states are bound as excitations take place predominantly on the Fe^+^ ion. At the lowest energies, five sextet terms and seven quartet terms lie close to each other, corresponding to the ^6^D([Ar]3d^6^4s) and ^4^F([Ar]3d^7^) atomic terms of Fe^+^, respectively. At the CCSD(T)/aug-cc-pVQZ//CCSD/aug-cc-pVTZ level, the sextet is more stable than the quartet by 0.01 eV, and the states are thus predicted to be isoenergetic within the expected computational error. Within about 1–3 eV, further 23 quartet states are found, several of them appear in the experimentally relevant region. Given the calculated energies, we can tentatively assign the feature observed in the 5000–6700 Å region as composed predominantly of the ^4^B_1_/^4^B_2_ terms, producing a broad, structureless band that is short-lived, possibly due to connection to other quartet terms or absorption of a second photon. The band at 4000–4900 Å would then correspond to excitation into the multitude of quartet electronic states starting at about 2.8 eV. The presence of a well-resolved vibrational structure about 4800 Å (2.6 eV) in the experimental spectrum suggests absorption into a bound quartet state; such a transition cannot be described within the reflection principle approximation applied here.

While we can assign the final states in the experiment as quartets, it is not fully clear whether predominantly sextet-quartet or quartet–quartet transitions are observed. Our simplified spectral modeling within the reflection principle (right-hand side of Figure 3) predicts that the sextet-quartet and quartet–quartet transitions have a cross-section on the order of 10^–23^ and 10^–20^ cm^2^, respectively. This clearly suggests that the majority of the experimental signal originates from quartet–quartet transitions. The sextet-quartet transitions, although possible, should not play a considerable role due to their low intensities.

Apart from the quartet states, states of doublet spin multiplicity start to appear close to 2.2 eV; they however exhibit low intensities in the spectrum. Further sextet states lie high in energy (above 3.3 eV), outside the experimentally studied range.

Since the excitations in the studied wavelength region involve only the 3d/4s electrons of Fe^+^, all accessible states are bound. Excitation into bound states with well-defined vibrational states leads to vibrational progressions in electronic absorption bands, which are broadened by their rotational envelope. This explains the sharp features in the spectrum, while the broad bands require either excitation to steep, repulsive sections of the potential energy surfaces or very efficient internal conversion to lower-lying electronic states leading to predissociation. The potential curves in Figure 3 represent only one of the six internal degrees of freedom of Fe^+^(H_2_O). The three vibrational modes of the H_2_O molecule will be largely spectators, but the in-plane and out-of-plane bending modes of the complex add substantial flexibility to the electronic states. At this point, we can speculate only that conical intersections are available to afford fast radiationless relaxation to low-lying electronic states, in which the available energy is sufficient for dissociation. As in the case of diatomic molecules such as CrO, MoO, or RhO studied by Morse and co-workers,^59^ the dense manifold of states displayed in Figure 3 may lead to a continuous photodissociation spectrum. Another possible photodissociation mechanism is the absorption of a second photon, which promotes an electron from the 3d to the 4p shell. Such a transition will have a 2–3 orders of magnitude higher cross section, so that absorption of a second photon within a 5 ns laser pulse has a high enough probability.

While the calculations successfully reproduce the overall spectral shape, it is not at all obvious what causes the structure in the spectra. It may be vibrational or rotational structure, but also the dense lying electronic states, especially after including spin–orbit coupling, could be responsible. Deuteration has been applied successfully to assign infrared spectra, e.g., of rare-gas tagged Co^+^(H_2_O).^61^ In the current scenario, repeating the experiment with Fe^+^(D_2_O) may not help since the shift in zero-point vibrational energy and Franck–Condon factors will severely affect the spectrum.

### Astrophysical Implications

The search for DIB carriers through laboratory spectroscopy has mostly focused on carbon compounds, especially PAHs, but only C_60_^+^ was identified as a DIB carrier.^10,11^ The identification of DIBs in the near-infrared region, however, requires even larger hydrocarbons to afford low-lying electronic transitions. Kappe et al.^12^ argued that larger PAHs are difficult to form and become less abundant as they grow more reactive. To be consistent with the observational data, DIB carriers must have strong absorption cross sections and high abundance. These two arguments taken together are somewhat at odds with PAHs as DIB carriers in the near-infrared. Despite all efforts, no progress has been made in the identification of DIB carriers since the C_60_^+^ work.^10^

Considering transition metals open the door to small molecules as DIB carriers, transitions in or from the open 3d shell of Fe or Fe^+^ easily cover the wavelength range of known DIBs. The required high abundances are plausible for strongly bound complexes of Fe^+^ composed of abundant elements, in particular, hydrogen, oxygen, and carbon. The relatively low binding energy of Fe^+^(H_2_O) of 1.33 eV^42^ and the weak 3d–3d transitions make this molecule in the end a weak candidate as a DIB carrier. Nevertheless, we learn from our experiment that sharp features can be found in the photodissociation spectrum on top of the broad absorption presumably caused by the dense manifold of electronic states. These features arise from excitations into bound states with a sufficient lifetime before internal conversion occurs so that a defined vibrational quantum state can manifest. We suggest that such a scenario could also be applied to DIBs. In this case, a broad, structureless band would be counted to the baseline of the observational spectrum (which would be hard to detect observationally), and the sharp features would be identified as the complete absorption originating from the DIB carrier. In general, broadening is caused by the rotational contour of the complex, together with the lifetime of the quantum state against radiationless relaxation processes, in particular, internal conversion or intersystem crossing. Fe^+^(H_2_O) is a typical prolate, near-symmetric top rotor, with a large rotational constant of 13.9 cm^–1^ for rotation about the Fe–O axis. For unperturbed electronically excited states, this leads to distinct rotational structure with peaks spaced by ≈ 28 cm^–1^ due to transitions with changes in K of ±1, as experimentally observed e.g. in the electronic spectra of Co^+^(H_2_O).^58^

Obtaining laboratory data for cationic iron compounds is difficult. Direct absorption spectroscopy, for example, cavity ringdown^62^ may work for neutral species, but the number density for ions is probably too small. The same holds true for laser-induced fluorescence, which has provided important spectra of neutral dimers embedded in molecular beams.^63^ Photodissociation spectroscopy works well for ions if the binding energy is smaller than the photon energy. Otherwise, multiple photons are necessary, which increases the experimental difficulty and reduces the signal levels. In addition, the experimental conditions may have a significant effect on the spectrum. Population of low-lying quantum states in a molecular beam may differ from the situation in interstellar clouds, where the molecules experience very few collisions, and their quantum state population is governed by absorption and emission of photons on much longer time scales. Therefore, additional peaks in a laboratory spectrum that are not present in observational data do not automatically rule out the molecule as a DIB carrier, as long as one or more bands are present that match. Also, the peak shape is heavily influenced by the population of rovibrational states in the laboratory experiment. Spectroscopy in helium droplets worked very well for C_60_^+^,^11^ but is limited to compounds that can be brought to a pickup cell, or can be formed in the droplet from species introduced via pickup cells.^12^

## Conclusions

Fe^+^ complexes have a multitude of excited states; therefore, one complex may be responsible for a significant number of DIBs. The spectra of Fe^+^(H_2_O) show that small complexes exhibit spectral features with the width and shape of DIBs. However, the mismatch in the band positions, the low absorption cross section, and the fact that the complex undergoes photodissociation ultimately make it a less favorable candidate as a DIB carrier. There is a certain chance that Fe^+^(H_2_O) turns out to be the carrier of DIBs in the near-infrared, but the probability for such a scenario is considered small. A more promising candidate is FeOH^+^. With a calculated binding energy of 3.70 eV, multiple-photon dissociation is required for photodissociation spectroscopy in the range relevant for DIBs, rendering such an experiment to be more challenging.

## Supporting Information Available

Additional computational details including benchmarks and full-range version of the potential curves (PDF)
Fe^+^(H_2_O) photodissociation spectrum (XLSX)
DIB spectrum toward HD 183143 (XLSX)
Computational data (XLSX)

## Footnotes

a http://archive.eso.org/cms/data-portal.html.
