# Supplementary material for: Iron Complexes as Potential Carriers of Diffuse Interstellar Bands: The Photodissociation Spectrum of Fe+(H2O) at Optical Wavelengths
Source: J Phys Chem A. 2024 Feb 13;128(7):1306–12. doi: 10.1021/acs.jpca.4c00148 (PMC10895653; doi:10.1021/acs.jpca.4c00148)
Supplement: Supplementary file 1 — jp4c00148_si_001.pdf [file jp4c00148_si_001.pdf]

Supporting Information

for

Iron complexes as potential carriers of diffuse interstellar bands: The photodissociation spectrum of $Fe^+(H_2O)$ at optical wavelengths

Marcos Juanes,[1,2] Shan Jin,[1] Rizalina T. Saragi,[1] Christian van der Linde,[1] Alexander Ebenbichler,[3] Norbert Przybilla,[3] Milan Ončák,[1] and Martin K. Beyer[1]

[1] Universität Innsbruck, Institut für Ionenphysik und Angewandte Physik, Technikerstraße 25, 6020 Innsbruck, Austria

[2] University of Valladolid, Dept. Química Física y Química Inorgánica, Paseo de Belén 7, 47011 Valladolid, Spain

[3] Universität Innsbruck, Institut für Astro- und Teilchenphysik, Technikerstraße 25, 6020 Innsbruck

1. Benchmarking of the multi-reference approach

In Tables S1 and S2, the sensitivity of electronic energies of sextet and quartet states on the active space size is analyzed. While the energies of the lowest sextet states are reasonably converged already with the smallest active space of (7,6), the quartet states are deeply influenced by the size of the active space, notably the 4^4B_1 and 4^4B_2 states in the experimental window.

Table S1 – Relative energies of electronic states calculated at the MRCI+Q/aug-cc-pVDz level in the most stable sextet state structure optimized at the CCSD/aug-cc-pVTZ level without inclusion of the spin-orbit coupling, using different active spaces. Active space description shows orbitals of irreducible representations in the (A_1, B_1, B_2, A_2) order. In the MRCI procedure, (7,2,2,0) core orbitals were employed. “*” marks usage of the aug-cc-pVTZ basis set.

active space	(7,6)	(7,7)	(7,8)	(7,9)	(7,9)	(7,9)*	(7,10)	(7,10)	(9,11)	(9,11)	(9,11)	(7,11)
closed-shell orbitals	(8,3,3,0)	(8,3,3,0)	(8,3,3,0)	(8,3,3,0)	(8,3,3,0)	(8,3,3,0)	(8,3,3,0)	(8,3,3,0)	(7,3,3,0)	(8,2,3,0)	(8,3,2,0)	(8,3,3,0)
active orbitals	(3,1,1,1)	(3,1,1,2)	(4,1,1,2)	(4,2,1,2)	(5,1,1,2)	(5,1,1,2)	(5,1,2,2)	(5,2,1,2)	(6,2,1,2)	(5,3,1,2)	(5,2,2,2)	(5,2,2,2)
1^6A_1	0.00	0.00	0.00	0.00	0.00	0.00	0.00	0.00	0.00	0.00	0.00	0.00
2^6A_1	0.40	0.39	0.39	0.39	0.39	0.41	0.39	0.39	0.40	0.34	0.30	0.39
1^6B_1	0.22	0.21	0.23	0.22	0.24	0.22	0.24	0.22	0.24	0.24	0.06	0.22
1^6B_2	0.11	0.10	0.12	0.12	0.13	0.13	0.13	0.12	0.14	-0.02	0.14	0.11
1^6A_2	0.01	0.00	0.01	0.01	0.00	0.01	0.00	0.01	0.00	0.05	0.02	0.00
1^4A_1	0.61	0.48	0.19	0.19	0.16	0.13	0.16	0.17	0.13	0.11	0.09	0.17
2^4A_1	1.28	1.28	1.24	1.22	1.21	1.17	1.21	1.21	1.19	1.13	1.18	1.21
3^4A_1	2.71	2.04	1.23	1.26	1.28	1.25	1.28	1.29	1.31	1.32	1.25	1.29
4^4A_1	3.10	3.10	3.01	3.01	3.03	2.94	3.03	3.02	3.02	2.96	2.94	3.01
5^4A_1	3.24	3.10	3.11	3.18	3.10	3.10	3.10	3.17	3.15	3.13	3.06	3.10
1^4B_1	0.86	0.38	0.39	0.34	0.29	0.24	0.29	0.32	0.27	0.23	0.19	0.28
2^4B_1	2.32	0.73	0.74	0.30	0.30	0.22	0.30	0.35	0.25	0.29	0.27	0.34
3^4B_1	2.40	1.66	1.66	1.00	1.05	0.94	1.05	1.04	1.04	1.03	0.94	1.03
4^4B_1	3.07	2.95	2.96	2.96	3.13	2.87	3.13	2.87	2.72	2.73	2.67	2.26
5^4B_1	3.15	3.08	3.09	3.00	3.19	2.92	3.19	3.05	3.05	3.01	2.97	2.96
1^4B_2	0.77	0.47	0.48	0.33	0.28	0.22	0.28	0.35	0.27	0.20	0.23	0.27
2^4B_2	2.26	0.67	0.69	0.35	0.29	0.27	0.29	0.27	0.21	0.27	0.21	0.33
3^4B_2	2.40	1.44	1.44	0.89	0.93	0.85	0.93	0.91	0.92	0.83	0.89	0.91
4^4B_2	3.07	2.97	2.98	2.97	3.10	2.88	3.10	3.00	2.99	2.86	2.96	2.15
5^4B_2	3.08	3.08	3.09	2.98	3.19	2.91	3.19	3.18	3.13	3.15	3.07	2.93
1^4A_2	0.62	0.24	0.25	0.21	0.16	0.16	0.16	0.17	0.13	0.11	0.10	0.18
2^4A_2	2.08	0.94	0.95	0.41	0.71	0.33	0.71	0.38	0.31	0.29	0.27	0.36
3^4A_2	2.74	1.23	1.23	1.24	1.20	1.21	1.20	1.22	1.20	1.24	1.22	1.22
4^4A_2	3.02	2.99	3.01	1.90	3.00	1.77	3.00	1.78	1.68	1.81	1.81	1.76
5^4A_2	3.10	3.05	3.06	3.04	3.10	2.98	3.10	3.16	3.13	3.12	3.04	2.92

Table S2 – Relative energies of electronic states calculated at the CASSCF/aug-cc-pVDZ level without inclusion of the spin-orbit coupling, using different active spaces. Active space description shows orbitals of irreducible representations in the (A_1, B_1, B_2, A_2) order.

active space	(7,11)	(7,12)	(7,12)	(7,12)	(9,12)	(9,13)	(9,13)	(9,13)	(7,13)	(7,13)	(7,13)	(7,14)	(7,16)	(9,15)
closed-shell orbitals	(8,3,3,0)	(8,3,3,0)	(8,3,3,0)	(8,3,3,0)	(8,2,3,0)	(8,2,3,0)	(8,2,3,0)	(8,2,3,0)	(8,3,3,0)	(8,3,3,0)	(8,3,3,0)	(8,3,3,0)	(8,3,3,0)	(8,2,3,0)
active orbitals	(5,2,2,2)	(6,2,2,2)	(5,3,2,2)	(5,2,3,2)	(5,3,2,2)	(6,3,2,2)	(5,4,2,2)	(5,3,3,2)	(6,3,2,2)	(6,2,3,2)	(5,3,3,2)	(6,3,3,2)	(7,4,3,2)	(6,4,3,2)
1^6A_1	0.00	0.00	0.00	0.00	0.00	0.00	0.00	0.00	0.00	0.00	0.00	0.00	0.00	0.00
2^6A_1	0.42	0.41	0.42	0.42	0.41	0.36	0.41	0.44	0.42	0.41	0.41	0.60	0.49	0.41
1^6B_1	0.22	0.22	0.22	0.22	0.22	0.15	0.21	0.23	0.22	0.22	0.27	0.32	0.29	0.23
1^6B_2	0.12	0.12	0.12	0.12	0.10	0.07	0.12	0.10	0.12	0.11	0.16	0.30	0.28	0.11
1^6A_2	0.00	0.00	0.01	0.00	0.00	-0.02	-0.01	-0.04	0.01	0.00	0.00	0.07	0.13	0.00
1^4A_1	0.42	0.46	0.43	0.43	0.42	0.41	0.35	0.43	0.47	0.47	0.36	0.42	0.27	0.47
2^4A_1	1.44	1.46	1.43	1.43	1.43	1.40	1.44	1.43	1.44	1.45	1.41	1.45	1.37	1.45
3^4A_1	1.51	1.52	1.49	1.49	1.51	1.48	1.49	1.55	1.50	1.50	1.47	1.68	1.57	1.50
4^4A_1	2.99	3.00	2.99	2.99	2.98	2.96	2.98	2.99	3.00	3.00	2.99	3.03	3.04	3.00
5^4A_1	3.06	3.07	3.07	3.07	3.04	3.03	3.04	3.03	3.08	3.08	3.06	3.18	3.16	3.07
1^4B_1	0.53	0.59	0.53	0.48	0.52	0.48	0.46	0.50	0.58	0.54	0.52	0.55	0.40	0.54
2^4B_1	0.58	0.64	0.55	0.60	0.58	0.55	0.54	0.57	0.61	0.65	0.58	0.62	0.50	0.66
3^4B_1	1.28	1.30	1.25	1.26	1.28	1.22	1.22	1.29	1.26	1.28	1.23	1.33	1.29	1.29
4^4B_1	2.68	2.73	2.59	2.68	2.67	2.63	2.62	2.69	2.64	2.73	2.70	2.71	2.55	2.73
5^4B_1	2.95	2.96	2.95	2.95	2.94	2.85	2.93	2.88	2.96	2.95	2.96	2.99	3.01	2.95
1^4B_2	0.53	0.58	0.54	0.46	0.52	0.50	0.42	0.54	0.59	0.52	0.51	0.52	0.44	0.52
2^4B_2	0.57	0.64	0.55	0.57	0.57	0.52	0.51	0.56	0.61	0.63	0.57	0.60	0.52	0.63
3^4B_2	1.17	1.19	1.14	1.15	1.16	1.12	1.17	1.15	1.16	1.17	1.13	1.30	1.26	1.17
4^4B_2	2.61	2.65	2.60	2.50	2.57	2.52	2.56	2.56	2.64	2.54	2.62	2.74	2.55	2.54
5^4B_2	2.92	2.93	2.92	2.91	2.89	2.74	2.90	2.80	2.93	2.92	2.93	2.96	3.00	2.91
1^4A_2	0.42	0.46	0.43	0.43	0.42	0.38	0.34	0.40	0.47	0.47	0.36	0.48	0.40	0.47
2^4A_2	0.61	0.67	0.57	0.57	0.60	0.53	0.54	0.62	0.63	0.63	0.62	0.63	0.53	0.63
3^4A_2	1.45	1.48	1.44	1.45	1.45	1.42	1.44	1.42	1.47	1.47	1.45	1.51	1.51	1.47
4^4A_2	2.21	2.26	2.20	2.20	2.21	2.19	2.13	2.21	2.25	2.25	2.19	2.10	2.06	2.25
5^4A_2	2.90	2.91	2.90	2.90	2.88	2.78	2.89	2.89	2.91	2.91	2.92	2.96	3.01	2.91

2. Details on spectra modeling

Due to the complicated electronic structure of the $Fe^{+}(H_2O)$ ion, several approximations were introduced for efficient molecular spectra modeling:

1) We employ reflection principle to model the spectra. Within this approximation, vibrational resolution of the spectra is lost.
2) Sampling of the potential energy surface is performed within harmonic approximation employing Monte Carlo integration of points arising from the Wigner distribution. The respective minimum structure and vibrational frequencies of the $Fe^{+}(H_2O)$ ion were calculated at the CCSD/aug-cc-pVTZ level of theory, corresponding to electronic states of $^4B_2$ and $^6A_2$ irreducible representations for quartet and sextet spin multiplicities, respectively. Note that further electronic states of other irreducible representations lie close in energy.
3) Only vibrational degrees of freedom of A_1 irreducible representation were picked, i.e. only three out of six vibrational degrees of freedom were considered, corresponding to symmetric O–H stretch, H_2O bending and Fe–OH_2 stretch.
4) The spectra were modeled using 200 points sampled from the Wigner distribution. In each point, an MRCI+Q(7,11) calculation including spin-orbit coupling was performed, with final spectrum using Gaussian broadening of calculated transitions with a full width at half maximum of 0.05 eV.

3. Cartesian coordinates (in Å) of optimized molecules and ions along with the zero-point energy (in Hartree) as optimized at the CCSD/aug-cc-pVTZ level

Fe+(H2O), M = 4
E = -1338.725949
Fe 0.000000 -0.000000 0.594765
O 0.000000 0.000000 -1.431226
H 0.000000 0.771055 -2.007042
H -0.000000 -0.771055 -2.007042

Fe+(H2O), M = 6
E = -1338.733073
Fe 0.000000 -0.000000 0.612739
O 0.000000 0.000000 -1.478032
H 0.000000 0.775592 -2.053478
H -0.000000 -0.775592 -2.053478

FeOH+, M = 5
E = -1338.125708
Fe 0.017796 -0.464565 0.000000
O 0.017796 1.261101 0.000000
H -0.605075 1.989871 0.000000

H2O
E = -76.312097
O -0.000000 0.000000 0.117558
H 0.000000 0.757635 -0.470233
H -0.000000 -0.757635 -0.470233

OH
E = -75.631141
O 0.000000 -0.000000 0.107859
H -0.000000 0.000000 -0.862870

Fe+, M = 4
E = -1262.3602599
Fe 0.000000 0.000000 0.000000

Fe+, M = 6
E = -1262.3704221
Fe 0.000000 0.000000 0.000000

Full-range version of the potential curves shown in Figure 3

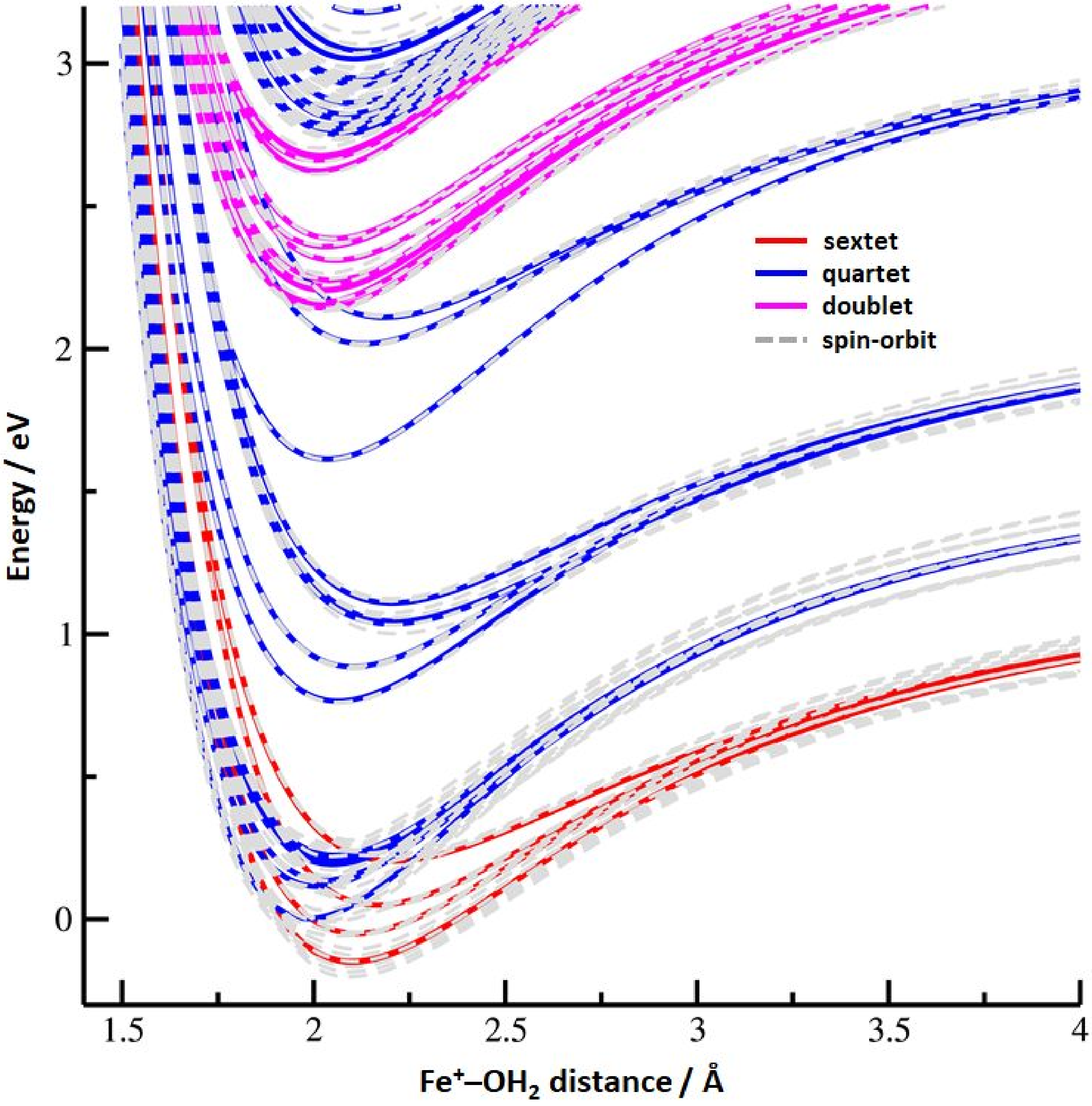

Figure S1: Potential energy curves for selected electronic states of $Fe^+(H_2O)$ along the $Fe^+...H_2O$ coordinate, with other structure parameters used as optimized at the CCSD/aug-cc-pVTZ level for quartet spin multiplicity. Splines are used to guide the eye.
